# Nomogram based on MRI features and clinical indicators for predicting the risk of limited mouth opening in patients with temporomandibular disorders

**DOI:** 10.1186/s12903-025-07519-5

**Published:** 2025-12-13

**Authors:** Chuanfang Xu, Xianyan Wu, Shibin Li, Qun Zhong, Jiena Pan, Chengbin Ye, Wenjie Yan

**Affiliations:** 1 Department of Radiology, The Affiliated People’s Hospital of Fujian University of Traditional Chinese Medicine, No. 602, 817 Middle Road, Taijiang District, Fuzhou, 350004 China; 2 Department of Rehabilitation, The Affiliated People’s Hospital of Fujian University of Traditional Chinese Medicine, Fuzhou, 350004 China

**Keywords:** Magnetic resonance imaging, Maximum mouth opening, Nomogram, Temporomandibular disorders, Temporomandibular joint

## Abstract

**Background:**

Limited mouth opening (LMO) is a cardinal manifestation of temporomandibular disorders (TMD). The prognostic value of magnetic resonance imaging (MRI) features for predicting LMO remains insufficiently defined. We aimed to systematically analyse MRI characteristics alongside clinical indicators in patients with TMD, identify independent predictors of LMO, and develop and validate an interpretable, clinically actionable nomogram.

**Methods:**

In this single-centre retrospective study, we included 584 consecutive patients clinically diagnosed with TMD according to the Research Diagnostic Criteria for Temporomandibular Disorders (RDC/TMD), who underwent MRI evaluation between January 2022 and December 2024, yielding 755 temporomandibular joints (TMJ). LMO was defined as maximum mouth opening (MMO) < 35 mm. Clinical features (age, sex) and MRI features (lesion side, disc position, disc morphology/signal/perforation, bilaminar zone tear, joint space, joint effusion, condylar movement, bony changes, lateral pterygoid muscle) were recorded. Candidate predictors were screened by univariable logistic regression and entered into multivariable logistic regression to identify independent predictors. A nomogram and risk-score model were constructed accordingly. The area assessed discrimination under the receiver-operating characteristic curve (AUC), model fit and calibration were evaluated using the Hosmer–Lemeshow test and calibration plots; clinical utility was examined with decision curve analysis (DCA).

**Results:**

Multivariate analysis revealed age, anterior disc displacement without reduction (ADDwoR), disc signal, and joint space as independent predictors of LMO. The resulting nomogram achieved an AUC of 0.762 (95% CI, 0.727–0.798), outperforming any single predictor or alternative combined model (AUC = 0.659; 95% CI, 0.617–0.700). The Hosmer–Lemeshow test and calibration plots indicated close agreement between the predicted and observed risks, and DCA demonstrated a positive net benefit across a broad range of clinically relevant thresholds.

**Conclusions:**

Age, ADDwoR, abnormal disc signal, and abnormal joint space jointly characterize the risk of LMO in the TMD. The proposed nomogram shows solid discrimination, calibration, and clinical utility, supporting first-visit risk stratification and individualized management. Prospective multicenter-centre studies with external validation are warranted to confirm generalizability.

**Supplementary Information:**

The online version contains supplementary material available at 10.1186/s12903-025-07519-5.

## Background

Temporomandibular disorders (TMD) are a common spectrum of conditions affecting the temporomandibular joint (TMJ) and surrounding tissues, with an adult prevalence of approximately 10–15% and a pronounced female predominance alongside a shift towards younger age groups [1–3]. The core symptoms include joint pain, clicking, masticatory dysfunction, and limited mouth opening (LMO). As a pivotal clinical manifestation, LMO directly compromises eating, speech, and social interaction, thereby challenging both quality of life and routine clinical management [1, 4]. Emerging evidence indicates that timely identification of LMO risk factors followed by effective intervention can meaningfully improve outcomes and patient-reported quality of life [5, 6].

Magnetic resonance imaging (MRI) is a preferred modality for characterizing structural abnormalities in patients with TMD. It delineates disc position, morphology, and signal alterations, and sensitively captures periarticular soft-tissue and osseous changes, thereby supporting diagnosis and disease assessment [7–9]. However, prior work has largely focused on single variables or limited feature sets, leaving a gap for integrative, multivariable prediction tools. In particular, models that combine MRI features with clinical indicators to estimate the risk of LMO remain scarce [10, 11].

Nomograms have gained broad traction for individualized risk stratification across clinical domains because they translate multivariable models into intuitive, point-based tools that quantify the contribution of each independent predictor and facilitate bedside decision-making [12–14]. To our knowledge, no study in the TMD field has reported a nomogram that jointly leverages MRI-derived features and clinical features to predict LMO risk.

Therefore, we aimed to systematically investigate MRI characteristics alongside clinical indicators in patients with TMD, identify independent predictors of LMO, and develop and validate an interpretable, clinically actionable nomogram. Our goal is to enable early recognition of high-risk individuals and to inform personalized management strategies in routine practice.

## Methods

### Study design and participants

This single-centre retrospective study included consecutive patients who presented to the Department of Rehabilitation Medicine at our institution between January 2022 and December 2024, and who were clinically diagnosed with TMD according to the Research Diagnostic Criteria for Temporomandibular Disorders (RDC/TMD) [22]. All clinical examinations were conducted by rehabilitation physicians at our institution, each with more than ten years of experience in TMD management. These physicians conducted standardized assessments in accordance with the RDC/TMD to ensure diagnostic consistency and reliability. MRI was subsequently performed as an imaging evaluation for RDC/TMD-confirmed patients, in accordance with the INfORM/IADR 2024 consensus, which recommends MRI only for specific clinical indications rather than for routine diagnosis [15, 16]. In total, 584 patients were enrolled, yielding 755 TMJs for analysis (413 unilateral and 171 bilateral cases).

The inclusion criteria were as follows: (1) clinical diagnosis established according to the standardized RDC/TMD; (2) complete clinical and MRI records; (3) no prior TMJ surgery; (4) no history of severe maxillofacial trauma; (5) no rheumatoid arthritis or other systemic diseases; and (6) MRI examination performed within one week after the clinical evaluation to ensure temporal consistency between clinical and imaging assessments. The exclusion criteria were as follows: (1) traumatic, neoplastic, infectious, postsurgical, or other organic TMJ lesions, as well as LMO caused by acute masticatory muscle spasm or other non-TMJ pathologies; (2) suboptimal MRI quality precluding diagnostic assessment; and (3) incomplete clinical or imaging data.

The study protocol was obtained from the Human Research Ethics Committee of The Affiliated People’s Hospital of Fujian University of Traditional Chinese Medicine (approval number: 2025-049−01), and written informed consent was waived owing to the retrospective design.

## Definition and assessment of LMO

LMO was assessed using maximum mouth opening (MMO) as an objective metric. With participants seated upright and the head–neck in a neutral position, trained researchers measured the vertical distance between the incisal edges of the upper and lower central incisors using a Vernier calliper, and recorded the maximum pain-free opening to the nearest millimetre. In accordance with published standards and clinical guidelines, MMO ≥ 35 mm was considered normal, whereas MMO < 35 mm was defined as LMO [21].

## MRI acquisition protocol

All TMJ MRI examinations were performed on two 3.0-T scanners (Siemens Verio, Siemens Healthineers, Germany; Philips Ingenia CX, Philips Healthcare, Netherlands). Oblique sagittal images were acquired parallel to the long axis of the mandibular condyle, and oblique coronal images were acquired perpendicular to that axis. The protocol included: (1) oblique sagittal PD-weighted imaging (PDWI) in closed- and open-mouth positions (Siemens: TR/TE = 3000/18 ms; slice thickness = 3 mm; field of view (FOV) = 150 × 150 mm; Philips: TR/TE = 2000/21 ms; slice thickness = 3 mm; FOV = 120 × 120 mm); (2) oblique sagittal T1-weighted imaging (T1WI) in closed-mouth position (Siemens: TR/TE = 600/9.4 ms; slice thickness = 3 mm; FOV = 150 × 150 mm; Philips: TR/TE = 450/8 ms; slice thickness = 3 mm; FOV = 120 × 120 mm); (3) oblique coronal PDWI in closed-mouth position (Siemens: TR/TE = 3000/18 ms; slice thickness = 3 mm; FOV = 150 × 150 mm; Philips: TR/TE = 2000/21 ms; slice thickness = 2 mm; FOV = 120 × 120 mm); (4) oblique coronal T2-weighted imaging (T2WI) in closed-mouth position (Siemens: TR/TE = 4200/40 ms; slice thickness = 3 mm; FOV = 150 × 150 mm; Philips: TR/TE = 3000/90 ms; slice thickness = 2 mm; FOV = 120 × 120 mm).

## Clinical and imaging features

Clinical features included age (years, continuous variable) and sex (male/female). All MRI features were evaluated according to rigorously predefined operational criteria to ensure consistency, reproducibility, and alignment with established TMJ imaging standards. The lesion side was recorded as left or right.

Disc position was classified into six categories according to predefined criteria: normal (posterior band located at the 12-o’clock position relative to the condyle in the closed-mouth position), anterior disc displacement with reduction (ADDwR; anterior displacement at mouth closure with return to the normal position upon opening), anterior disc displacement without reduction (ADDwoR; persistent anterior displacement during opening), medial displacement (disc positioned medial to the condylar head on coronal images), lateral displacement (disc positioned lateral to the condyle on coronal images), and disc adhesion (disc exhibiting a fixed appearance with restricted or absent translational movement). The disc morphology was categorized as normal (biconcave configuration) or abnormal (flattened, folded, or otherwise deformed). A disc signal was considered abnormal when focal or diffuse hyperintensity was present on PDWI or T2WI. Disc perforation was defined as a focal discontinuity of the disc contour accompanied by an adjacent fluid-like signal. Bilaminar zone tears were diagnosed when irregular, disrupted, or heterogeneous high-signal changes were observed in the retrodiscal tissues. The joint space was rated as normal or abnormalities, with abnormality defined as narrowing, widening, or asymmetry of the superior or posterior compartments. Joint effusion was considered present when hyperintense fluid accumulation was visible within the joint cavity on T2-weighted or PD-weighted images. Condylar movement was classified as normal (condyle translating to the articular eminence), restricted (translation not reaching the eminence), or excessive (translation extending beyond the eminence apex). Bony changes were categorized as normal or abnormal, with abnormalities including cortical flattening, erosion, osteophyte formation, or subchondral sclerosis. The lateral pterygoid muscle was assessed as normal or abnormal; abnormalities were defined as morphological alterations, hypertrophy, atrophy, or heterogeneous T2 hyperintensity.

Diagnostic criteria for all MRI features were defined according to established references on temporomandibular joint imaging, primarily following Bag et al. and Aiken et al. [7, 8]. Two radiologists with 6 and 7 years of experience in TMJ MRI independently evaluated all the imaging features following the standardized workflow and operational definitions described above. Discrepant cases were flagged for a second joint review, during which both readers reexamined the images together on the same workstation and discussed each item according to the predefined diagnostic criteria. If disagreements persisted after discussion, a senior radiologist with 12 years of experience in TMJ MRI acted as an adjudicator and provided the final determination. The adjudicated ratings were recorded as the final reference standard for subsequent analyses.

## Standardization of imaging assessment

To ensure methodological consistency and to minimize interreader variability, all MRI assessments were performed using a standardized workflow. Before data extraction, all the readers participated in a calibration session in which 30 representative TMJ MRI scans were jointly reviewed. During this session, the two readers discussed the operational definitions of each MRI features, refined the interpretation thresholds, and aligned their understanding of the rating criteria until full procedural agreement was reached.

All the imaging evaluations were performed using a unified PACS workstation with identical monitor specifications and standardized window width and window level settings. A structured scoring sheet incorporating all operational definitions was used to guide the assessment of every case, ensuring that each features was documented according to the same criteria. Readers were fully blinded to the clinical information, symptom severity, and study outcomes throughout the evaluation process.

After calibration, the two readers independently assessed all MRI examinations following the standardized procedure without further interaction. This harmonized workflow ensured that each imaging feature was rated under consistent viewing conditions and according to the same predefined criteria, thereby reducing potential subjective bias and enhancing reproducibility. Inter-observer agreement was evaluated using Cohen’s κ (weighted κ for ordinal variables) in a randomly selected subset of 100 TMJs joints.

### Statistical analysis

Analyses were performed using SPSS 26.0 (IBM, Armonk, NY, USA) and R 4.4.3 (R Foundation for Statistical Computing, Vienna, Austria). Continuous variables are reported as the mean ± standard deviation (SD) and categorical variables are reported as counts (percentages). Candidate predictors of LMO were first screened with univariable logistic regression (two-sided *P* < 0.05). Variables meeting these criteria were entered into multivariable logistic regression to identify independent predictors; odds ratios (ORs) with 95% confidence intervals (CIs) are reported. For each independent predictor, we generated univariable receiver operating characteristic (ROC) curves to assess individual discrimination. In parallel, we fitted a combined prediction model and plotted its ROC curve to evaluate overall discrimination for LMO.

A nomogram was then derived from the final multivariable model using the rms package in R, converting regression coefficients into a point-based scoring system for individualized risk estimation. Discrimination was quantified by the area under the curve (AUC) with 95% CI. Model fit and calibration were assessed with the Hosmer–Lemeshow (H-L) test and internally validated using bootstrap resampling (1,000 resamples) to generate calibration curves and compare the predicted risk with the observed risks thereby mitigating overfitting. Clinical utility was examined with decision curve analysis (DCA) by calculating the net benefit across a range of threshold probabilities and comparing the model with “treat-all” and “treat-none” strategies. Two-sided *P* < 0.05 was considered to indicate statistical significance.

## Results

### Baseline characteristics: LMO vs. non-LMO

**Fig. 1:**
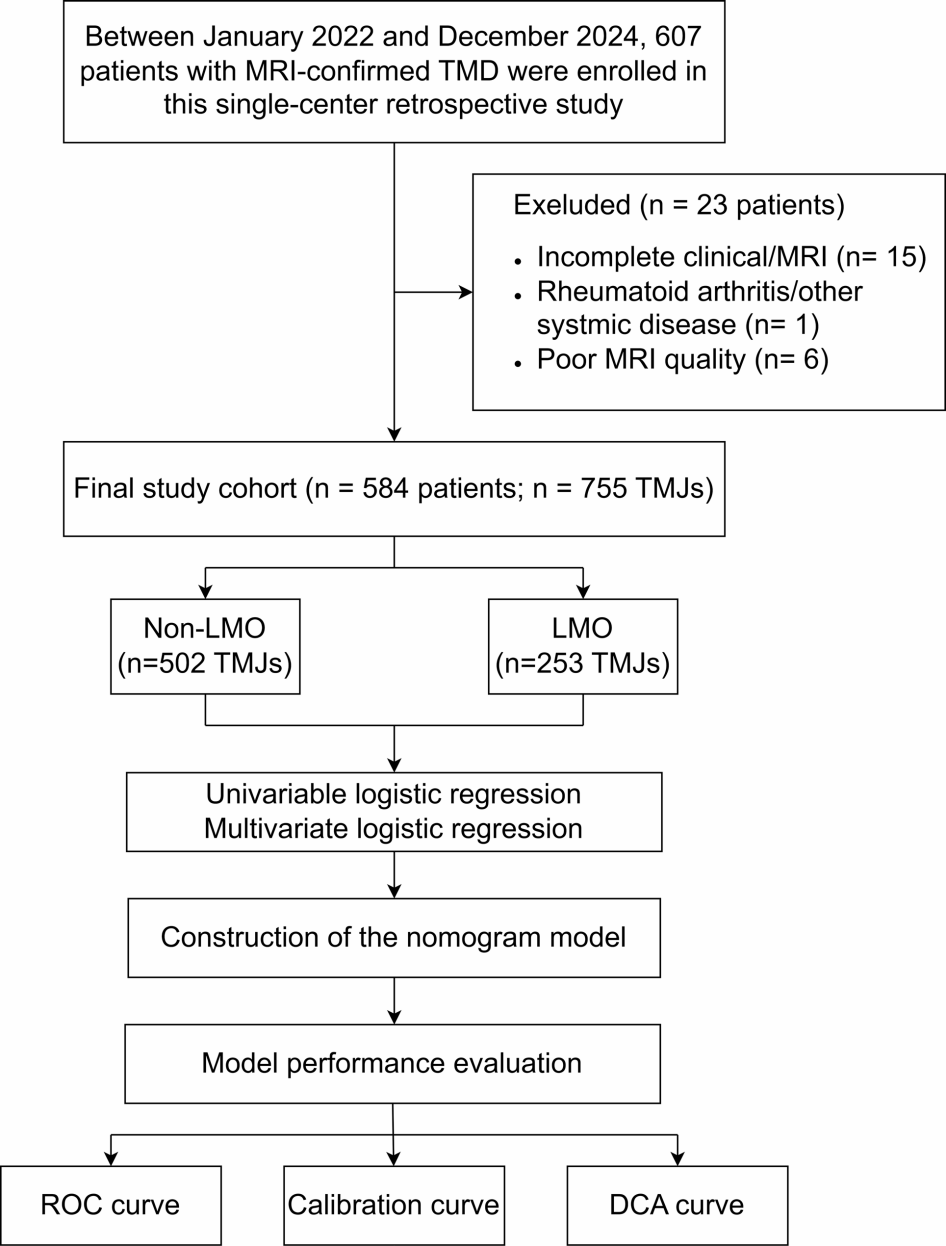
Study flowchart: Patient selection and analysis workflow

The study flowchart illustrating the patient selection and analysis workflow is presented in Fig. 1. A total of 584 patients contributed 755 TMJs, of whom 253 (33.5%) met the LMO criterion and 502 (66.5%) did not meet the LMO criterion. Patients in the LMO group were significantly older than those in the non-LMO group (*P* < 0.001). The proportion of males was modestly greater in the LMO group (*P* = 0.047).

In terms of MRI features, the LMO group had higher frequencies of abnormal disc position (*P* = 0.001), abnormal disc morphology (*P* < 0.001), abnormal disc signal (*P* < 0.001), and abnormal joint space (*P* = 0.004). The lesion side, disc perforation, bilaminar zone tear, joint effusion, condylar movement, bony changes, and lateral pterygoid muscle did not significantly differ between the groups (all *P* > 0.05; Table 1).

**Table 1:** Baseline clinical and MRI features of the TMD cohort

Characteristic	Non-LMO (*n* = 502)	LMO (*n* = 253)	t/χ^2^/Z values	*P*-values
Age	27.71 ± 10.73	32.75 ± 15.01	−5.307	<0.001
Sex			3.929	0.047
Female	101(20.1%)	36(14.2%)		
Male	401(79.9%)	217(85.8%)		
Lesion side			1.046	0.306
Left	266(53.0%)	144(56.9%)		
Right	236(47.0%)	109(43.1%)		
Disc position				0.001
Normal	159(31.7%)	55(21.7%)		
ADDwR	202(40.2%)	93(36.8%)		
ADDwoR	131(26.1%)	101(39.9%)		
Medial displacement	1(0.2%)	0(0)		
Lateral displacement	1(0.2%)	0(0)		
Disc adhesion	8(1.6%)	4(1.6%)		
Disc morphology			13.836	<0.001
Normal	406(80.9%)	174(68.8%)		
Abnormal	96(19.1%)	79(31.2%)		
Disc signal			16.229	<0.001
Normal	474(94.4%)	217(85.8%)		
Abnormal	28(5.6%)	36(14.2%)		
Disc perforation				1.000
Yes	501(99.8%)	253(100.0%)		
No	1(0.2%)	0(0)		
Bilaminar zone tear			3.286	0.070
Yes	489(97.4%)	240(94.9%)		
No	13(2.6%)	13(5.1%)		
Joint space			8.151	0.004
Normal	459(91.4%)	214(84.6%)		
Abnormal	43(8.6%)	39(15.4%)		
Joint effusion			0.017	0.897
Yes	308(61.4%)	154(60.9%)		
No	194(38.6%)	99(39.1%)		
Condylar movement			4.804	0.091
Normal	296(59.0%)	143(56.5%)		
Restricted condylar movement	151(30.1%)	92(36.4%)		
Excessive condylar movement	55(11.0%)	18(7.1%)		
Bony changes			0.116	0.733
Normal	310(61.8%)	153(60.5%)		
Abnormal	192(38.2%)	100(39.5%)		
Lateral pterygoid muscle			0.009	0.925
Normal	274(54.6%)	139(54.9%)		
Abnormal	228(45.4%)	114(45.1%)		

### Inter-observer agreement

Inter-observer agreement was evaluated in a randomly selected subset of 100 temporomandibular joints independently assessed by both radiologists. Cohen’s κ values ranged from 0.662 to 0.951, indicating substantial to almost perfect agreement across all MRI features. Disc perforation showed the lowest agreement (κ = 0.662), consistent with previous TMJ MRI literature due to its low prevalence and subtle imaging appearance. All other MRI features demonstrated almost perfect agreement (κ = 0.823–0.951). Detailed κ statistics for each feature are provided in Supplementary Table S1.

### Univariable and multivariable logistic regression

On univariable analyses, age (*P* < 0.001), ADDwoR (*P* = 0.029), abnormal disc signal (*P* < 0.001), and abnormal joint space (*P* = 0.014) were significantly associated with LMO. Sex, lesion side, disc morphology, joint effusion, condylar movement, bony changes, and lateral pterygoid muscle were not significantly associated with LMO (all *P* > 0.05).

**Fig. 2:**
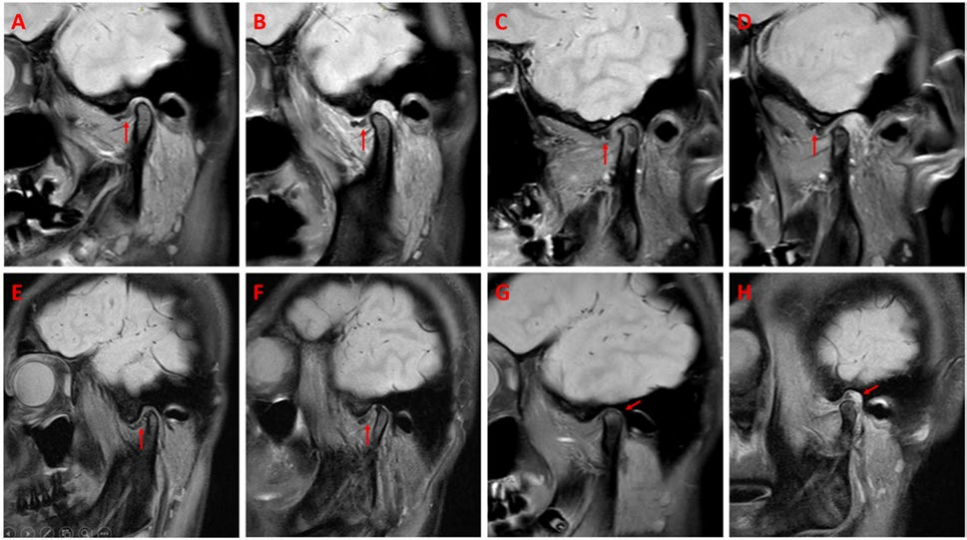
Representative MRI features associated with LMO in TMD patients. **A**, **B** Case 1 of ADDwoR. **A** Closed-mouth MRI showings anterior disc displacement (red arrow); (**B**) Open-mouth MRI demonstrating persistent nonreduction (red arrow). **C**, **D** Case 2 of ADDwoR. **C** Closed-mouth MRI showing anterior disc displacement (red arrow); (**D**) Open-mouth MRI demonstrating persistent nonreduction (red arrow). **E**, **F** Abnormal disc signal (red arrows). **G**, **H **Abnormal joint space. **G** Narrowed joint space (red arrows); (**H**) Widened joint space (red arrows).

Multivariable logistic regression confirmed that age (*P* < 0.001), ADDwoR (*P* = 0.001), abnormal disc signal (*P* < 0.001), and abnormal joint space (*P* = 0.017) were independent predictors of LMO (Table 2; Fig. 2).

**Table 2:** Univariable and multivariable logistic regression analysis of clinical and MRI features for predicting LMO in TMD patients

Clinical and MRI features	Univariable logistic regression		Multivariable logistic regression		
	OR(95%CI)	*P*-values		OR(95%CI)	*P*-values
Age	1.031 (1.018–1.044)	<0.001		1.030 (1.018–1.043)	<0.001
Sex(Female vs. Male)	1.182 (0.762–1.835)	0.455			
Lesion side(Left vs. Right)	0.837 (0.606–1.154)	0.278			
Disc position					
Normal					
ADDwR	1.287 (0.845–1.961)	0.239			
ADDwoR	1.765 (1.061–2.938)	0.029		2.019 (1.333–3.058)	0.001
Medial displacement	0 (0–0)	1.000			
Lateral displacement	0 (0–0)	1.000			
Disc adhesion	1.381 (0.372–5.128)	0.630			
Disc morphology abnormal	1.225 (0.788–1.904)				
Disc signal abnormal	2.863 (1.634–5.014)	<0.001		2.889 (1.685–4.951)	<0.001
Disc perforation	0 (0–0)	1.000			
Bilaminar zone tear	1.110 (0.454–2.713)	0.818			
Joint space abnormal	1.863 (1.131–3.067)	0.014		1.814 (1.111–2.961)	0.017
Joint effusion	0.905 (0.647–1.266)	0.561			
Condylar movement					
Normal					
Restricted condylar movement	1.361 (0.949–1.952)	0.094			
Excessive condylar movement	0.711 (0.386–1.313)	0.276			
Bony changes abnormal	0.776 (0.548–1.099)	0.154			
Lateral pterygoid muscle abnormal	1.012 (0.728–1.407)	0.945

**Fig. 3:**
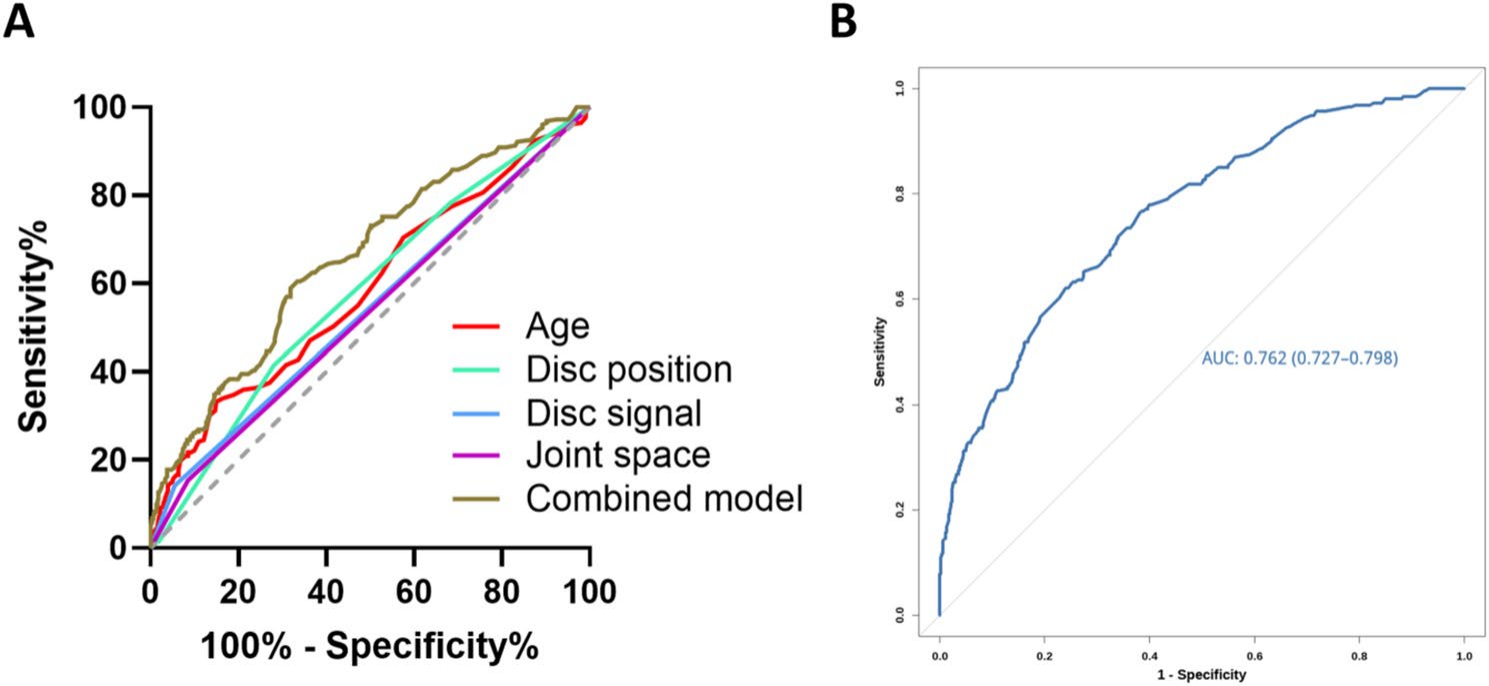
ROC curves of clinical and MRI predictors for estimating the risk of LMO in TMD patients. **A** ROC curves for individual predictors (age, ADDwoR, disc signal, and joint space) and for the combined model. The corresponding AUCs were 0.587 (95% CI, 0.543–0.631), 0.580 (95% CI, 0.537–0.623), 0.543 (95% CI, 0.499–0.588), 0.534 (95% CI, 0.490–0.579), and 0.659 (95% CI, 0.617–0.700), respectively. **B** ROC curve of the final nomogram model integrating age, disc position, disc signal, and joint space. The nomogram achieved an AUC of 0.762 (95% CI, 0.727–0.798), demonstrating superior discrimination compared with single predictors or the simple combined model

The results of the ROC analysis of individual predictors revealed modest discrimination: age (AUC = 0.587; 95% CI, 0.543–0.631), disc position (AUC = 0.580; 95% CI, 0.537–0.623), disc signal (AUC = 0.543; 95% CI, 0.499–0.588), and joint space (AUC = 0.534; 95% CI, 0.490–0.579). A combined logistic model incorporating these four predictors yielded an AUC of 0.659 (95% CI, 0.617–0.700), representing a modest improvement over any single variable (Fig. 3 A).

### Nomogram construction and performance

**Fig. 4:**
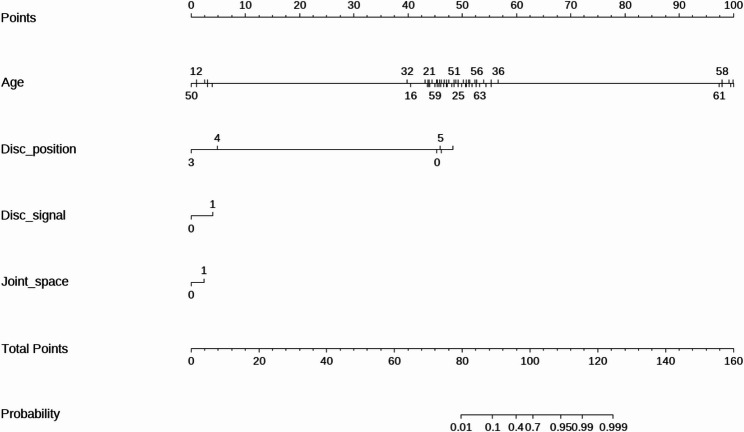
A nomogram incorporating four independent predictors—age, ADDwoR, disc signal, and joint space—was developed to estimate the risk of LMO in patients with TMD

**Fig. 5:**
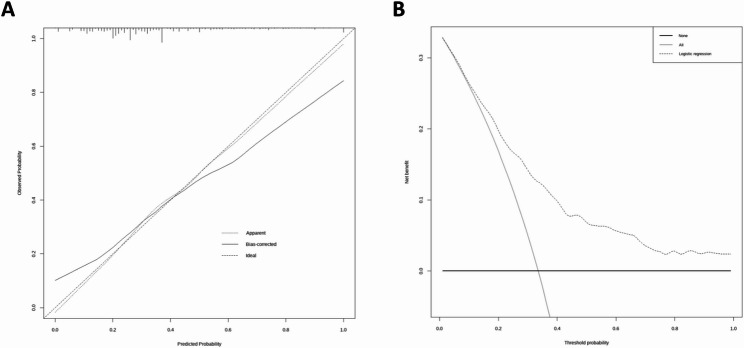
Calibration and decision curve analyses of the nomogram model for predicting the risk of LMO in patients with TMD. **A** Calibration curve of the nomogram. The apparent and bias-corrected curves show close agreement between the predicted and observed probabilities, demonstrating good model calibration. **B** DCA of the nomogram. The model yields a positive net benefit across a broad range of threshold probabilities compared with the “treat-all” and “treat-none” strategies, supporting its clinical utility for individualized risk assessment

A nomogram integrating the four independent predictors (age, ADDwoR, abnormal disc signal, and abnormal joint space) was developed to estimate the patient-level risk of LMO (Fig. 4). This model demonstrated robust discrimination with an AUC of 0.762 (95% CI, 0.727–0.798), outperforming all the single predictors and the simple combined model (Fig. 3B). Calibration revealed close agreement between the predicted and observed risks, with a mean absolute error of 0.031 (Fig. 5 A). The H-L test indicated no lack of fit (χ² = 4.859, *P* = 0.773). DCA (Fig. 5B) demonstrated a positive net benefit across a broad range of clinically relevant threshold probabilities, exceeding all-treatment or no-treatment strategies and supporting the model’s utility for individualized risk stratification and clinical decision-making.

## Discussion

Leveraging MRI features alongside clinical indicators, a nomogram was developed and validated to estimate the risk of LMO in patients with TMD. Age, ADDwoR, abnormal disc signal, and abnormal joint space emerged as independent predictors. The model demonstrated strong discrimination (AUC = 0.762; 95% CI, 0.727–0.798), good calibration, and consistent net clinical benefit across decision thresholds, underscoring its potential utility for risk assessment and stratified management in routine care.

Among the imaging markers, ADDwoR was strongly associated with the LMO (multivariable OR = 2.019), highlighting its central role in restricting mandibular excursion. During mouth opening, failure of the displaced disc to recapture the condyle can create a mechanical block, alter mandibular kinematics, and directly constrain the range of motion [13]. Prior MRI studies align with these findings: Yasan et al. [26] reported substantially reduced MMO and a marked increase in LMO risk among patients with ADDwoR compared with those with reducible displacement or normal disc position; Özel et al. [23] similarly reported a higher incidence of ADDwoR in LMO, often accompanied by disc deformity and osteoarthritic changes. Proposed mechanisms include tension within retrodiscal tissues, disordered intra-articular pressure, and reactive effusion—processes that collectively limit joint mobility and accelerate degenerative trajectories [20–22].

An abnormal disc signal independently predicted LMO (OR = 2.889). This MRI feature plausibly reflects reduced hydration, matrix degeneration, or inflammatory activity within the disc [23, 24]. Empirical evidence further links disc signal alterations with disc degeneration and condylar marrow oedema, conditions that frequently coexist with restricted mandibular function [25, 26]. As such, an aberrant disc signal may serve as an imaging surrogate for tissue injury and an early warning sign for functional compromise.

An abnormal joint space was also associated with LMO (OR = 1.814). Joint space disturbance can index disc–condyle incongruity, chondral degeneration, or anterior condylar positioning, each of which may disrupt lubrication, redistribute load, and reduce excursion capacity [27, 28]. Although alterations in disc signal intensity and joint space morphology may represent secondary degenerative changes following ADDwoR, they were considered in this study as imaging indicators of cumulative structural impairment relevant to functional limitations. When present alongside ADDwoR and abnormal disc signals, joint-space abnormalities may amplify a coupled “mechanical imbalance–inflammatory response–degeneration” pathway, explaining their stable contribution to the multivariable model [21, 29]. From a practical standpoint, joint-space alterations on MRI should be regarded as a salient red flag for LMO risk.

Beyond imaging, age emerged as an independent predictor (OR = 1.030). Age-related reductions in tissue elasticity and hydration, progressive cartilage wear, and osteophyte formation diminish joint compliance and increase resistance to motion, collectively heightening the risk of functional limitation [7]. Older individuals may also present a greater burden of ADDwoR and degenerative stigmata, thereby compounding the likelihood of LMO [19]. Similarly, population-based data show that MMO decreases with age, in parallel with increased clicking, which is consistent with an age-linked biomechanical and structural pathway to restricted opening [11]. Moreover, as Yadav et al. [29] noted, some age-related morphological changes in the TMJ may reflect physiological remodelling rather than overt pathology, although reduced tissue elasticity and adaptability with ageing can still predispose individuals to LMO.

By integrating age with three pathophysiologically anchored MRI features (disc position, abnormal disc signal, and abnormal joint space) the nomogram provides an interpretable, actionable tool for individualized risk estimation. Its performance exceeded that of any single predictor (AUCs 0.534–0.587) and a simple combined model (AUC = 0.659; 95% CI, 0.617–0.700), indicating that LMO risk is not dominated by a single feature but rather arises from the confluence of structural and tissue-level alterations. The results of the calibration analyses revealed close agreement between the predicted and observed risks (mean absolute error = 0.031; H-L test *P* = 0.773), and bootstrap validation supported the model stability. Compared with all-treatment or no-treatment strategies, DCA demonstrated consistent net benefit across clinically relevant thresholds, supporting its use for first-visit triage, early intervention planning, and longitudinal restratification during follow-up.

In a previous study, Özel et al. [23] analyse 46 patients with TMD using 3 T MRI and reported that LMO was primarily associated with ADDWoR, disc degeneration, and disc deformation, highlighting degenerative changes as a fundamental cause of functional impairment. In comparison, our study, which included a substantially larger cohort of 755 temporomandibular joints, demonstrated that LMO was not only strongly correlated with ADDWoR, but also significantly associated with age, abnormal disc signal intensity, and joint space alterations. These findings suggest that in addition to disc position and degenerative morphology, patient age and MRI-detectable alterations in disc and joint space characteristics play critical roles in the development of LMO. By expanding upon earlier observations, our results emphasize the multifactorial nature of TMD-related functional restriction and underscore the value of large-scale cohorts in elucidating the complex aetiological mechanisms underlying impaired mandibular mobility. Unlike the screening-focused nomogram by Cui et al. [6], which targeted TMD risk in a university population using questionnaire/psychosocial metrics (e.g., DC/TMD) without imaging, our study addresses a clinical TMD cohort and a functionally defined endpoint (LMO). By explicitly incorporating MRI features that map to disc–condyle imbalance, soft-tissue mobility, and mechanical obstruction, our model offers stronger pathophysiologic specificity and clinical interpretability for decision-making in symptomatic patients Strengths include a clearly defined clinical outcome, standardized MRI acquisition across 3.0-T platforms, and dual-reader assessment with consensus resolution. Nevertheless, several limitations merit attention. First, the single-centre retrospective design, despite coverage of 755 joints, introduces risks of selection and information bias; prospective multi-centre studies are needed to confirm generalizability. Second, although expert readers assessed the images, interpretive subjectivity cannot be fully eliminated. Future work should incorporate radiomics and deep learning to extract reproducible, high-dimensional features and to quantify the incremental value of AI-enhanced modelling for functional prediction. Third, psychosocial variables (e.g., stress and anxiety), which can modulate pain and muscle function, were not included. The incorporation of such measures with structural imaging may enhance both the performance and interpretability of predictive models. Given the multifactorial nature of TMD and LMO, imaging-based modelling should be interpreted as an adjunct to comprehensive evaluation rather than as a stand-alone diagnostic approach. Future studies should integrate psychosocial and behavioural indicators alongside imaging biomarkers within a person-centred management framework to achieve a more holistic understanding of TMD pathophysiology and functional limitations. Finally, while internal calibration was robust, external validation and, if needed, recalibration in independent cohorts remain essential for establishing transportability across centres and scanners.

## Conclusions

This study identifies age, ADDwoR, abnormal disc signal, and abnormal joint space as key, independent correlates of LMO in TMD patients and presents a parsimonious nomogram with favourable discrimination, calibration, and clinical net benefit. This tool supports early screening and dynamic risk stratification, informs individualized treatment pathways, and provides a practicable framework for future multimodal predictive research that integrates quantitative imaging and patient-centred factors.

## Supplementary Information

Supplementary Material 1

## Data Availability

The datasets generated and/or analyzed during the present study are not publicly available due to patient privacy restrictions but can be obtained from the corresponding author upon reasonable request.
