# Supplementary material for: Nomogram based on MRI features and clinical indicators for predicting the risk of limited mouth opening in patients with temporomandibular disorders
Source: BMC Oral Health. 2025 Dec 13;26:120. doi: 10.1186/s12903-025-07519-5 (PMC12821808; doi:10.1186/s12903-025-07519-5)
Supplement: Supplementary file 1 — Supplementary Material 1 [file 12903_2025_7519_MOESM1_ESM.docx]

**Supplementary Table S1. Inter-observer agreement for MRI features**

| **MRI Features** | **Cohen’s κ** | **Agreement Level*** |
| --- | --- | --- |
| Disc position | 0.903 | Almost perfect |
| Disc morphology | 0.904 | Almost perfect |
| Disc signal | 0.911 | Almost perfect |
| Disc perforation | 0.662 | Substantial |
| Bilaminar zone tear | 0.823 | Almost perfect |
| Joint space alteration | 0.918 | Almost perfect |
| Joint effusion | 0.951 | Almost perfect |
| Condylar movement | 0.903 | Almost perfect |
| Bony changes | 0.899 | Almost perfect |
| Lateral pterygoid muscle | 0.855 | Almost perfect |

***Agreement Level was interpreted according to the Landis and Koch criteria: < 0.00 = poor; 0.00–0.20 = slight; 0.21–0.40 = fair; 0.41–0.60 = moderate; 0.61–0.80 = substantial; 0.81–1.00 = almost perfect.
